# The estimation of per capita loadings of domestic wastewater in Tehran

**DOI:** 10.1186/s40201-015-0174-2

**Published:** 2015-03-24

**Authors:** Alireza Mesdaghinia, Simin Nasseri, Amir Hossein Mahvi, Hamid Reza Tashauoei, Mahdi Hadi

**Affiliations:** Center for Water Quality Research (CWQR), Institute for Environmental Research (IER), Tehran University of Medical Sciences, Tehran, Iran; Department of Environmental Health Engineering, Faculty of Health, Tehran University of Medical Sciences, Tehran, Iran; Center for Solid Waste Research (CSWR), Institute for Environmental Research (IER), Tehran University of Medical Sciences, Tehran, Iran; Department of Environmental Health Engineering, School of Public Health, Islamic Azad University-Tehran Medical Branch, Tehran, Iran

**Keywords:** Domestic wastewater, Per capita loading, Wastewater treatment plant

## Abstract

The amount of wastewater characteristics loading is one of the main parameters in the design of wastewater collection and treatment systems. The generation per capita per day (GPCD) of wastewater characteristics was estimated by analyzing the monthly data of nine wastewater treatment plants in Tehran, capital city of Iran. GPCD values were calculated from measured collected wastewater flow, the population and concentration data. The results indicated the values of 32.96 ± 1.91, 49.25 ± 2.49, 37.31 ± 2.44, 6.77 ± 0.53, 1.96 ± 0.11, 92.23 ± 5.68, 2.07 ± 0.39 and 128.96 ± 6.69 g/d.cap of GPCD for BOD_5_, COD, TSS, TKN, P, TDS, ON and TS, respectively, for Tehran’s wastewater. The per capita estimated for the wastewater production and treatment were determined to be 186.06 ± 7.85 and 136.72 ± 5.43 L/d.cap, respectively. It is estimated that about 504 m^3^/d and 346 m^3^/d of sludge, will be produced and waste as excrement raw sludge, respectively, in Tehran. Simple regression models were presented the relationships such as the change of collected and treated wastewater with population and changes of GPCD parameters with each other. It was revealed that the Tehran’s wastewater may be classified as highly degradable, but during recent decades its Biodegradability Index (BI) has been reduced up to 15%. The new suggested revised per capita parameters can be used for design purposes in Tehran, and possibly, in areas with similar characteristics, substituting the classical values obtained from foreign textbooks. These values could help in designing more accurate treatment systems and may lower the required capacity for the treatment of wastewater up to 40% in Tehran.

## Introduction

The amount of wastewater characteristics loading is one of the main parameters in the design of wastewater collection and treatment systems. The per capita loading of wastewater characteristics such as chemical oxygen demand (COD), Biological oxygen demand (BOD_5_), nitrogen, phosphorus and solids have been considered as useful main functions in the design of wastewater collection systems and in the control of water resources pollution [1].

The pollutants per capita values can be used to estimate the present and the future pollution loading of wastewater produced from a population. These also are useful to estimate the equivalent population of an urban or industrial wastewater flow [2]. By expressing the wastewater pollution in terms of per capita values, the concept of pollution would be more understandable for the citizens and policy makers. However, the changes in mode and living standards [3] and the development of wastewater collection and treatment technologies, would suggest that a review and a re-examination of the pollution per capita loadings be made [1]. On the other hand, the discharge of these pollutants can cause considerable problems in environment [4]. Increased pollution of water-receiving bodies, and the imposition of restrictive limits by local administrations, led a need for new treatment technologies [5-9]. Thus it is very important that the properties of the discharged wastewater be assessed in order to be able to improve current technologies and provide adequate wastewater treatment [10].

Tehran, the capital city of Iran, has a population of over 7.5 million. There are nine public and 18 private wastewater treatment plant (WWTP) in Tehran those can treat more than 100 MCM of wastewater per year [11]. By construction of a new treatment plant system in the south of Tehran which can treat about 750 million cubic meters per year after project completion, the other treatment systems will be switched off gradually. Based on the current existing information of in-use domestic wastewater treatment plants in Tehran it may be possible to make an estimate of wastewater per capita parameters that can be used for design purposes and development programs of wastewater treatment systems in Tehran.

One of aims of the study reported in this paper is to re-establish the main wastewater pollution measures (including BOD_5_, COD, TSS, TKN, P, TDS, ON(Organic Nitrogen) and TS) generation per capita per day (GPCD) according to the recorded data of nine public wastewater treatment systems in Tehran. The per capita loading of wastewater is an important parameter in the design process of a treatment plant’s units. Iran’s water and sewage utility (ABFA company) states that per capita water consumption in Tehran is currently about 378 liters per day [11]. Our study also sought to determine the amount of wastewater produced per capita per day or the conversion factor of water to wastewater. Pollutant discharge per capita (PDC; g/d.cap) with the wastewater treatment system in Tehran is another parameter that is important in the case of wastewater discharge in to receiving bodies. This parameter and the per capita of the producing sludge for domestic wastewater in Tehran would also be estimated in this study from wastewater treatment plants data. The later parameter may be useful in sludge treatment and management programs. The findings of this study can be used as basic data for the design of wastewater treatment systems in Tehran and possibly in areas with similar characteristics.

## Materials and methods

The data were used in this study obtained from monthly reports (from 2007 to 2013) of nine wastewater treatment plants in Tehran including Sahebgharaniyeh, Mahallti, Zargandeh, Qeitariyeh, Qods, Shush, Ekbatan, Dowlatabadi and Jonoob. The data includes the quantitative information of wastewater production and characteristics data over 80 months from April 2007 to November 2013 for all domestic WWTPs in Tehran. The analysis of wastewater samples were done by the laboratory stuffs of WWTPs according to Standard Methods for the Examination of Water and Wastewater [12]. The parameters monitored were temperature, pH, Biological Oxygen Demand (BOD_5_), Total Dissolved Solid (TDS), Total Solids (TS), Total Suspended Solid (TSS), Chemical Oxygen Demand (COD), Organic Nitrogen (ON), Total Phosphorous (TP) and Total Kjeldahl Nitrogen (TKN). Data analysis involved the data pre-processing and conducting some descriptive and analytical studies using Microsoft Excel 2007 and R software packages.

The study included data pre-processing and preparing them to make the estimates of desired parameters. Initial data processing, although is a time consuming step, but is very important part in the success of statistical analysis [13]. At this step, the raw data of nine in-use WWTPs collected during the recent years was assessed. The raw data was included 720 instances, although there were some outlier values. The data that appear to be very distant from the normal data distribution may be classified as being outliers. In certain instances however, this outlying value may be correct and is a natural product of the variables distribution [14]. All examples with missing values were represented with NA (not available). In this study, we took a normal distribution with a cutoff of three times of standard deviations around the mean to detect the outliers. Thus, the data that was more than μ ± 3SD was considered as outliers.

The descriptive statistics of raw (720 instances) and pre-processed (499 instances) data are summarized in Tables 1 and 2, respectively.

**Table 1:** **The statistics of raw data of wastewater characteristic for Tehran WWTPs**

**Parameter**	**Mean**	**SD**	**SE**	**Max.**	**Min.**	**UB**	**LB**
Nomin	300670	281473	24756	900000	10500	325427	275914
Collect	458191	807439	85130	7445021	39000	543321	373060
Treat	397265	796648	83993	7445021	11395	481258	313272
ProSlu	88.16	77.63	6.83	225.00	1.00	94.99	81.34
ExcSlu	64.15	57.37	5.05	188.00	1.00	69.20	59.11
CurentPop	90558	172233	18159	1459808	7000	108717	72399
Tin	21.83	3.81	0.42	29.50	12.90	22.24	21.41
pHin	7.96	0.34	0.04	9.00	6.91	7.99	7.92
BODin	171.85	54.42	5.93	352.00	15.00	177.78	165.92
CODin	259.60	79.69	8.74	507.00	28.80	268.34	250.87
TSin	697.97	171.35	19.03	1280.00	246.00	716.99	678.94
TSSin	198.65	69.09	7.61	400.00	30.00	206.26	191.04
TDSin	494.88	145.66	17.72	985.00	185.00	512.60	477.16
Pin	11.24	4.68	0.53	29.70	3.92	11.77	10.72
OrNin	11.30	6.05	1.99	28.80	0.97	13.29	9.31
TKNin	35.16	12.33	1.55	79.38	12.00	36.71	33.61
Tout	20.94	4.13	0.45	28.20	10.00	21.39	20.49
pHout	7.39	0.35	0.04	9.00	6.50	7.43	7.35
BODout	11.90	8.96	0.98	72.28	2.40	12.88	10.93
CODout	23.95	12.83	1.41	98.50	6.40	25.36	22.54
TSout	482.38	135.12	15.03	934.50	3.00	497.41	467.35
TSSout	14.25	14.04	1.55	114.00	1.00	15.80	12.70
TDSout	465.12	128.18	15.59	902.00	214.00	480.71	449.52
Pout	4.24	1.60	0.18	12.97	0.05	4.42	4.06
OrNout	0.93	0.75	0.09	5.00	0.00	1.02	0.83
TKNout	4.28	4.33	0.57	23.85	0.04	4.85	3.71

SD, Standard deviation; SE, 1.96 × Standard error; Max., Maximum of observation; Min., Minimum of observation; UB, Upper bound of 95% confidence interval; LB, Lower bound of 95% confidence interval.

**Table 2:** **The statistics of pre-processed data of wastewater characteristic for Tehran WWTPs**

	**Mean**	**SD**	**SE**	**Max.**	**Min.**	**UB**	**LB**
Nomin	300670	281473	24757	900000	10500	325427	275914
Collect	458190	807439	85130	7445021	39000	543321	373060
Treat	397265	796648	83993	7445021	11395	481258	313273
ProSlu	88.16	77.63	6.83	225.00	1.00	94.99	81.34
ExcSlu	64.15	57.37	5.05	188.00	1.00	69.20	59.11
CurentPop	90558	172233	18159	1459808	7000	108717	72399
Tin	21.83	3.81	0.42	29.50	12.90	22.24	21.41
pHin	7.96	0.34	0.04	9.00	6.91	7.99	7.92
BODin	171.85	54.42	5.93	352.00	15.00	177.78	165.92
CODin	259.60	79.69	8.74	507.00	28.80	268.34	250.87
TSin	697.97	171.35	19.03	1280.00	246.00	716.99	678.94
TSSin	198.65	69.09	7.61	400.00	30.00	206.26	191.04
TDSin	494.88	145.66	17.72	985.00	185.00	512.60	477.16
Pin	11.24	4.68	0.53	29.70	3.92	11.77	10.72
OrNin	11.30	6.05	1.99	28.80	0.97	13.29	9.31
TKNin	35.16	12.33	1.55	79.38	12.00	36.71	33.61
Tout	20.94	4.13	0.45	28.20	10.00	21.39	20.49
pHout	7.39	0.35	0.04	9.00	6.50	7.43	7.35
BODout	11.90	8.96	0.98	72.28	2.40	12.88	10.93
CODout	23.95	12.83	1.41	98.50	6.40	25.36	22.54
TSout	482.38	135.12	15.03	934.50	3.00	497.41	467.35
TSSout	14.25	14.04	1.55	114.00	1.00	15.80	12.70
TDSout	465.12	128.18	15.59	902.00	214.00	480.71	449.52
Pout	4.24	1.60	0.18	12.97	0.05	4.42	4.06
OrNout	0.93	0.75	0.09	5.00	0.00	1.02	0.83
TKNout	4.28	4.33	0.57	23.85	0.04	4.85	3.71

SD, Standard deviation; SE, 1.96 × Standard Error; Max., Maximum of observation; Min., Minimum of observation; UB, Upper bound of 95% confidence interval; LB, Lower bound of 95% confidence interval.

The estimation of pollutant generation per capita per day (GCPD) values was conducted according to the following equations:

$$ \begin{array}{l}GCP{D}_{BOD5} = \left(\left( Collect/30\right) \times BODin\right)/ CurentPop\hfill \\ {}GCP{D}_{COD} = \left(\left( Collect/30\right) \times CODin\right)/ CurentPop\hfill \\ {}GCP{D}_{TSS} = \left(\left( Collect/30\right) \times TSSin\right)/ CurentPop\hfill \\ {}GCP{D}_{TKN} = \left(\left( Collect/30\right) \times TKNin\right)/ CurentPop\hfill \\ {}GCP{D}_P = \left(\left( Collect/30\right) \times Pin\right)/ CurentPop\hfill \\ {}GCP{D}_{TDS} = \left(\left( Collect/30\right) \times TDSin\right)/ CurentPop\hfill \\ {}GCP{D}_{OrgN} = \left(\left( Collect/30\right) \times OrNin\right)/ CurentPop\hfill \\ {}GCP{D}_{TS} = \left(\left( Collect/30\right) \times TSin\right)/ CurentPop\hfill \\ {}GCP{D}_{Slu\_P} = \left(\left( ProSlu/30\right)\ / CurentPop\right) \times 1000\hfill \\ {}GCP{D}_{Slu\_E} = \left(\left( ExcSlu/30\right)\ / CurentPop\right) \times 1000\hfill \\ {} Col{l}_{PerP} = \left(\left( Collect/ CurentPop\right)/30\right) \times 1000\hfill \\ {}Tre{t}_{PerP} = \left(\left( Treat/ CurentPop\right)/30\right) \times 1000\hfill \end{array} $$

The pollutant discharge per capita (PDC; g/d.cap) with the wastewater treatment system was defined and determined in terms of pollutant generation per capita (GCPD; g/d.cap) and pollutant removal efficiency (PRE; %) of the wastewater treatment systems as follow [15]:

$$ PDC = GCPD \times \left[\left(100-PRE\right)/100\right] $$

## Results and discussion

**Figure 1:**
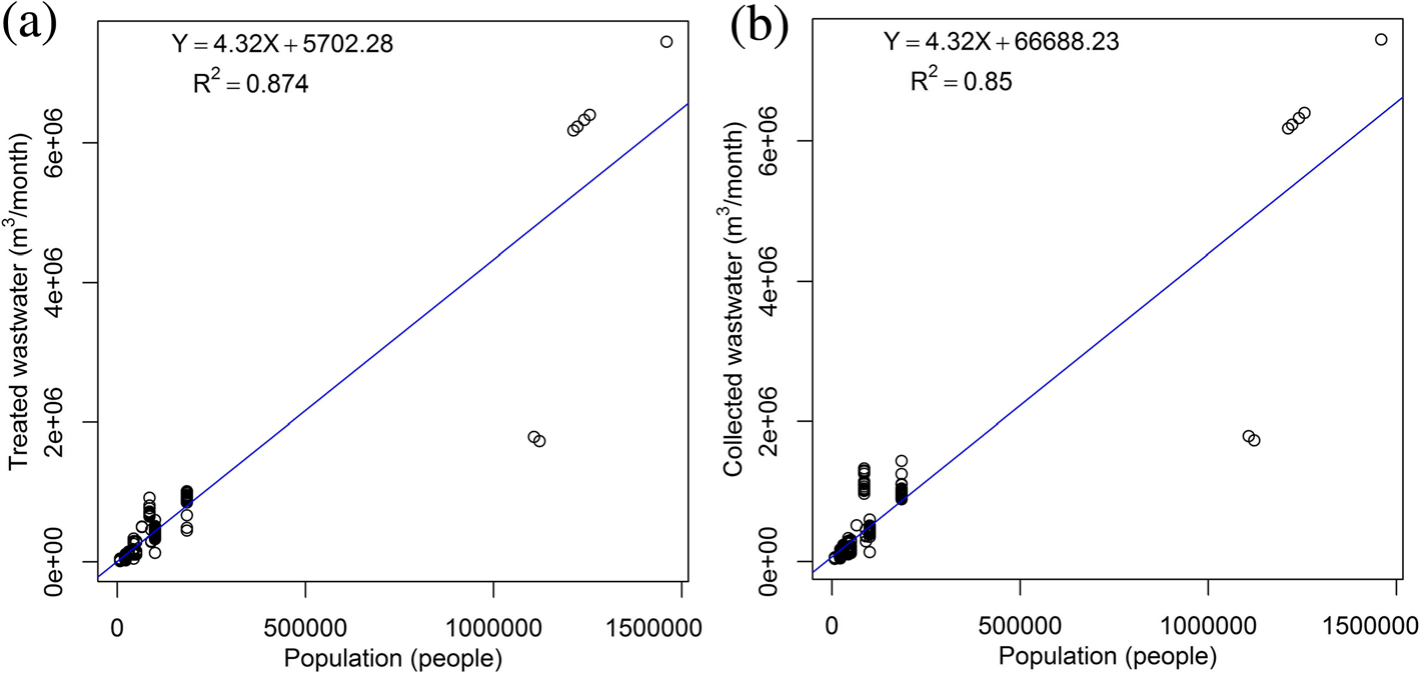
**The flow of treated (a) and collected (b) wastewater versus population.**

The total yearly averaged population covered by Tehran’s nine municipal wastewater treatment plants from 2007 to 2013 was determined to be 4,502,065 persons per year. In average, out of 22,778,632 m^3^/year estimated collected wastewater from 2007 to 2013, only 19,749,770 m^3^/year of it were treated. In other words, about three MCM per year (13% of estimated collected wastewater from 2007 to 2013) were discharged into the environment without adequate treatment. The mathematical relationships between wastewater flows and population in Tehran were shown in Figure 1.

According to the latest report of the basic operational items of Tehran’s wastewater company [16], a capacity of 675,000 m^3^/d had been allocated for the treatment of wastewater produced by 3,150,000 persons up to June 2014. This means that the per capita loading of wastewater considered by designers for the design of a wastewater treatment plant in Tehran, is about 215 L/d. By a roughly estimation according to Figure 1(b), it is expected that for a population of 3,150,000 persons, the capacity of 455,822 m^3^/d is a sufficient capacity that needs to be allocated. This capacity is about 40% lower than current allocated value. Therefore, the overestimated per capita loading value of 215 L/d in Tehran, leads the system to be designed for a capacity more than that is required.

The pattern changes of Tehran wastewater quality contents from 1984 to 2013 is another issue were assessed in Table 3. The most of the wastewater quality parameters have not shown considerable changes during this period. However, averaged value of TSS parameter in the influent wastewater of Tehran’s WWTPs was considerably decreased from 353.33 in 1984 to 198.65 mg/L in 2013. It is clear that TSS content of Tehran domestic wastewater has been decreased by 56% of its value in 1984. This result indicates that the per capita generation of TSS parameter could also be reduced similarly.

**Table 3:** **Changes of Tehran wastewater quality contents from 1984 to 2013**

**Year**	**Parameters**							**Ref.**
	**pH**	**BOD**_**5**_	**COD**	**TSS**	**TDS**	**P**	**TKN**	
**1984**	n.a.	288.33	n.a.	353.33	495	25	n.a.	[17]
**1993**	7.43	184.47	261.87	256.03	n.a.	n.a.	45.56	[18]
**1995**	7.39	170	237.8	226.3	n.a.	9.9	39.37	[19]
**1997**	7.8	129.8	225	189.7	n.a.	2.7	38.3	[20]
**2013**	7.96	171.85	259.6	198.65	494.88	11.24	35.16	This study

n.a: not available.

The estimated per capita loadings for domestic wastewater of Tehran, as summarized from the results of this investigation, are presented in Table 4. The GPCD values of 32.96 ± 1.91, 49.25 ± 2.49, 37.31 ± 2.44, 6.77 ± 0.53, 1.96 ± 0.11, 92.23 ± 5.68, 2.07 ± 0.39 and 128.96 ± 6.69 g/d.cap were estimated for BOD_5_, COD, TSS, TKN, P, TDS, ON and TS, respectively. In a study conducted by Azimi and Ameri [20] on the estimation of per capita loadings for domestic wastewater of Saheb-Gharanieh treatment plant in Tehran (based on the data collected in 1997), they were found GPCD values of 36, 62, 52, 10.5 and 0.74 g/d.cap for BOD_5_, COD, TSS, TKN and P, respectively. Although their estimated values are not representative of all treatment plants, comparison of them with the GPCD estimated in our study show that the GPCD of BOD_5_, TKN, and P were not considerably changed, but it was decreased by 71% and 79% of estimated values (in 1997) for TSS and COD parameters, respectively. This result is consistent with the pattern of TSS changes in Table 3.

**Table 4:** **The estimated values of per capita parameters**

**Parameter**	**Mean**	**SD**	**SE**	**Max.**	**Min.**	**UB**	**LB**
GCPD_Slu_E_	0.11	0.14	0.02	0.89	0.00	0.12	0.09
GCPD_Slu_P_	0.16	0.23	0.02	1.07	0.00	0.19	0.14
GCPD_TS_	128.96	56.39	6.69	395.04	40.07	135.65	122.27
GCPD_OrgN_	2.07	1.18	0.39	5.76	0.17	2.45	1.68
GCPD_TDS_	92.23	43.03	5.68	303.75	27.98	97.90	86.55
GCPD_P_	1.96	0.91	0.11	5.60	0.53	2.07	1.85
GCPD_TKN_	6.77	3.88	0.53	31.14	2.19	7.30	6.24
GCPD_TSS_	37.31	20.76	2.44	160.44	3.20	39.76	34.87
GCPD_COD_	49.25	21.25	2.49	140.06	5.76	51.73	46.76
GCPD_BOD5_	32.96	16.41	1.91	104.07	2.82	34.86	31.05
(BOD_5_/COD)_out_	0.41	1.06	0.12	7.25	0.95	2.51	2.27
(BOD_5_/COD)_in_	0.61	1.20	0.13	21.87	0.23	1.75	1.49
Tret_PerP_	136.72	51.53	5.43	360.75	33.61	142.15	131.28
Coll_PerP_	186.06	74.42	7.85	522.71	44.83	193.91	178.22
PDC_TS_	89.49	38.92	4.63	283.05	0.39	94.12	84.86
PDC_OrgN_	1.82	0.92	0.32	4.03	0.17	2.14	1.49
PDC_TDS_	87.32	36.07	4.77	279.52	29.75	92.09	82.55
PDC_P_	0.76	0.29	0.04	1.60	0.25	0.80	0.73
PDC_TKN_	0.73	0.84	0.12	4.88	0.01	0.85	0.61
PDC_TSS_	2.47	2.33	0.28	21.54	0.26	2.75	2.20
PDC_COD_	4.46	2.93	0.34	25.51	0.84	4.81	4.12
PDC_BOD_	2.15	1.60	0.19	15.31	0.36	2.34	1.96

SD, Standard deviation; SE, 1.96 × Standard Error; Max., Maximum of observation; Min., Minimum of observation; UB, Upper bound of 95% confidence interval; LB, Lower bound of 95% confidence interval.

For evaluating the biodegradability of Tehran’s domestic wastewater the BOD_5_/COD ratio, is called Biodegradability Index (BI), was determined. The BI Index varies from 0.4 to 0.8 for domestic wastewaters. If BOD_5_/COD is > 0.6 then the waste is fairly biodegradable and can be effectively treated biologically. If BOD_5_/COD ratio is between 0.3 and 0.6, then seeding is required to treat it biologically. If BOD_5_/COD is < 0.3 then it cannot be treated biologically [21,22]. From data in the literature (Table 3) and the results of this study, the BI index was obtained to be 0.70, 0.71, 0.57 and 0.61 for the years of 1993, 1995, 1997 and 2013, respectively. Although these values reveal that the wastewater may be classified as highly biodegradable, BI values for later two years are almost 15% lower than those for previous years. This may be resulted from this fact that over the past 15-20 years a wide range of diverse industrial synthetic detergents which are mostly made from petroleum products and alcohols, has been produced and extensively used for cleaning and disinfection purposes and then discharged into sewage systems [23]. Many detergent compounds was found to be resistant to biodegradation by bacteria [24]. This may be the main reason of deceasing in the biodegradation potential of Tehran’s wastewater according to the BI index.

Table 5 summarizes the mathematical simple relationships among the estimated GCPD parameters. The strongest relationship was found between GCPD_COD_ and GCPD_BOD_ (R^2^ = 0.74). The next relationship with high correlation (R^2^ = 0.66) was found between GCPD_BOD_ and GCPD_TSS_. These relationships may be useful for designers to help them to estimate the GPCD values from each other.

**Table 5:** **The main relationships between Tehran wastewater quality parameters**

**X**	**Y**	**Equation**	**R** ^**2**^
GCPD_COD_	GCPD_BOD_	Y = 0.66X + 0.16	0.74
GCPD_BOD_	GCPD_TSS_	Y = 1.03X + 3.61	0.66
GCPD_COD_	GCPD_TSS_	Y = 0.76X-0.15	0.60
GCPD_BOD_	GCPD_TDS_	Y = 2.03X + 25.88	0.58
GCPD_COD_	GCPD_TDS_	Y = 1.55X + 16.55	0.56
GCPD_BOD_	GCPD_TKN_	Y = 0.17X + 1.17	0.52
GCPD_COD_	GCPD_TKN_	Y = 0.12X + 0.62	0.45
GCPD_TSS_	GCPD_TKN_	Y = 0.12X + 2.31	0.42
GCPD_COD_	GCPD_P_	Y = 0.03X + 0.72	0.35
GCPD_BOD_	GCPD_P_	Y = 0.03X + 0.92	0.33
GCPD_TSS_	GCPD_P_	Y = 0.02X + 1.16	0.30

The per capita collected wastewater (Coll_PerP_) was determined to be 186.06 ± 7.85 while the Coll_PerP_ of 199.67 L/d.cap was estimated from the study’s results of Sharifi Sistani [25]. This finding shows that this parameter has been decreased in 2013 by 93% of its value in 2000. This seven percent decrease during recent thirteen years may be results from some reasons such as increasing the level of public awareness on water saving tips, using of treated water for construction purposes and cars or yards washing and probably increased use of fast foods instead of cooking meals at home.

Table 6 compares the per capita values of wastewater quality parameters estimated for Iran’s domestic wastewater treatment plants in 2001. These values are drawn from a report of Department of Energy [26]. As shown in this table, different cities in Iran have different values for per capita loadings.

**Table 6:** **Basic characteristics of raw sewage intended for the design of wastewater treatment plants in Iran** [26]

**Paramater**	**Per capita parameters as g/d.cap for different plant or cities**					
	**Toiserkan**	**Zargandeh (Tehran)**	**Parkandabad (Mashhad)**	**Hovaizeh**	**Arak**	**Zabol**
BOD_5_	40	40	50	54	57	57
TSS	50	83	46	50	60	50
N(TKN)	-	14	-	-	5	-
TP	-	1	-	-	0.45	

As shown in Table 6 the GCPD values of BOD_5_, TSS, TKN and TP in 2001, according only to one wastewater treatment plant data (Zargandeh), were more than those for 2013 obtained to be 28.9, 32.3, 6.1 and 1.6 g/d.cap, respectively. The result indicates and confirms that the GCPD of TSS considerably reduced during the recent decade.

According to the technical criteria standard N. 3-129 [27], a new sewage treatment plant in Iran should be designed at least based on a per capita average of 40 to 50 g/d.cap BOD_5_ and 50 to 60 g/d.cap of total suspended solids (TSS). These values have been universally used in the design of wastewater treatment systems and unchallenged since the publication of this standard in Iran. The recommended values of 40-50 g/d.cap for BOD_5_ and 50-60 g/d.cap for TSS are more than the values of 31.05-34.86 and 34.87-39.76 g/d.cap estimated in this study, respectively. In another word, the estimated values of GCPD for BOD_5_ and TSS for Tehran’s wastewater are 27% and 32% lower than the mean of recommended values by Department of Energy, respectively. Using the guideline values recommended by Department of Energy for the design of wastewater treatment plant for the city of Tehran, with different living habits in different parts of the city, may results in considerable overestimations in the design process of new treatment plants. Thus, it is recommended that our estimated GPCDs be used in the design of new wastewater treatment plants in Tehran instead of recommended values by Department of Energy.

In Table 7, the GCPD values for main wastewater parameters in different countries were compared with the estimated GCPD values for Tehran’s wastewater. The estimated values for Tehran are close to the per capita values obtained for countries such as Turkey, India, and Egypt. In these countries, the GCPD values are lower than that for European countries and United States. Low consumption of toilet papers in this countries and especially in Tehran may be one of the reasons for the low values of GCPD for BOD_5_ and TSS [20].

**Table 7:** **Comparison of per capita loadings (g/d.cap) of wastewater parameters in different countries with estimated values in Tehran**

**Parameter**	**BOD5**	**TSS**	**TKN**	**TP**
Turkey^a^	27-50	41-68	8-14	0.4-2
India^a^	27-41	n.a.	n.a.	n.a.
Japan^a^	40-45	n.a.	1-3	0.2-0.4
Egypt^a^	27-41	41-68	8-14	0.4-0.6
Uganda^a^	55-68	41-55	8-14	0.4-0.6
Italy^a^	49-60	55-82	8-14	0.6-1
Germany^a^	55-68	82-96	11-16	1.2-1.6
Denmark^a^	55-68	82-96	14-19	1.5-2
Sweden^a^	68-82	82-96	11-16	0.8-1.2
Brazil^a^	55-68	55-68	8-14	0.6-1
United States^a^	50-120	60-150	9-22	2.7-4.5
Tehran (This study)	31-34	35-40	6.2-7.3	1.8-2

a: Adapted from *Wastewater engineering: treatment, disposal, and reuse* [28].

It is estimated that over 90% of the treated wastewater effluent from treatment plants across Iran country is reused in some way; however, much of it is mixed with freshwater before further use, particularly in the suburban areas [29]. The direct use of untreated wastewater from sewage outlet, directly used for crop production is not a common scene in Iran. However, treated or partially treated wastewater used directly for irrigation without being mixed or diluted is more common. This is practiced in many treatment plants and there is no exact estimate about the amount used by this method to irrigate fodders, cereals, fruit trees, and vegetables eaten cooked or uncooked [29]. Thus the pollutants discharge per capita (PDC; g/d.cap) with the wastewater treatment systems is important parameter should be considered to be estimated.

The PDC with the wastewater treatment systems in Tehran was defined and determined in terms of GCPD and pollutant removal efficiency (PRE; %). The pollutants removal efficiency of wastewater treatment systems in Tehran ranged 92.11–93.41% for BOD_5_, 89.65–90.97% for COD, 91.48–93.18% for TSS, 28.65-31.72% for TS, 1.73-2.76% for organic nitrogen (ON), 58.52-61.24% for P and 84.62-88.26% for TKN.

The ranges of estimated PDC summarized in Table 4, were compared with conventional domestic wastewater treatment systems in the United States [30] (Table 8). The generation per capita of BOD_5_, COD, ON and TP of the United States wastewater treatment systems were almost thrice, four times, nine times and twice as those in Tehran, respectively. Conventional and nutrient removal activated sludge treatment systems are regarded as representative wastewater treatment processes in urban area of developed countries such as USA. PDCs with these treatment systems were estimated as 16.1 g-BOD_5_/d.cap, 31.6 g-COD/d.cap, 4.9 g-ON/d.cap, and 3.2 g-TP/d.cap [30] while PDCs with Tehran’s wastewater treatment systems were estimated as 2.15 ± 0.19 g-BOD_5_/d.cap, 4.46 ± 0.34 g-COD/d.cap, 1.82 ± 0.32 g-ON/d.cap, and 0.76 ± 0.04 g-TP/d.cap. Deployment of larger removal efficiency treatment systems will decrease PDC with increase of PRE. When judging only from pollutant discharge reduction function of treatment systems, countries or cities with smaller GCPD and larger PDC-BOD_5_ should be prioritized for investments on wastewater treatment measurements including assistances in order to improve ambient water quality [31].

**Table 8:** **Comparison of GDPCs and PDCs of conventional activated sludge wastewater treatment systems in the USA** [30] **with those in Tehran**

**Country**	**Parameters**	**BOD5**	**COD**	**ON**	**TP**
United States (Qasim, 1998)	GDPC (g/d.cap)	95	180	18	4
	Removal efficiency (%)	80-85	80-85	60-85	10-25
	PDC (g/d.cap)	16.1	30.6	4.9	3.2
Tehran (This study)	GDPC (g/d.cap)	31.0-34.8	46.7-51.7	1.6-2.4	1.8-2.0
	Removal efficiency (%)	92.1-93.4	89.6-90.9	1.7-2.7	58.5-61.2
	PDC ((g/d.cap)	1.96-2.3	4.1-4.8	1.4-2.1	0.7-0.8

The estimated PDC of some regions studied by Tsuzuki [15] were compared with that of Tehran and summarized in Table 9. When the criteria are applied, the group of countries in the Region of South China Sea and Tehran were found to be with higher and lower priority of BOD_5_ discharge reduction from domestic wastewater, respectively (Table 9). The PDC of ROPME^a^ sea area region, Red sea region and Gulf of Aden region is 2.5 g/d.cap and comparable with that obtained for Tehran (2.15 g/d.cap).

**Table 9:** **Comparison of PDC of other regions from 1998 to 2002** [15] **with that of Tehran**

**Region/country**	**PDC (g/d.cap)**	
	**BOD**	**TP**
South China Sea Region	43	n.a
Caspian Sea Region	24	n.a
Eastern African Region	11	n.a
Pacific Island Region	8.5	0.51
West and Central African (WACAF) Region	4.6	1.8
ROPME^a^ sea area and Red sea and gulf of Aden region	2.50	1.10
Tehran (this study)	2.15	0.76

a: Regional Organization for the Protection of the Marine Environment.

The comparatively low BOD_5_-PDC in sewage treatment plants of Tehran may results from the fact that the per capita loading of BOD_5_ in Tehran is lower than that for other countries and on the other hand the removal efficiency of BOD_5_ in most of treatment plants are more than 90%.

Tajrishi [29] estimated that less than 40% of the total domestic sludge in Iran’s wastewater treatment plants is being treated completely. In other words; of more than 200,000 cubic meters of daily produced sludge (2000 tons/d dry solids) of total fecal, septic and waste excrements sludge, only about 80,000 cubic meters (800 tons) is being digested and/or stabilized daily by different treatment methods. According to the finding of our study, the per capita values of 0.16 ± 0.02 and 0.11 ± 0.02 L sludge/d were determined for the produced and waste excrements sludge in Tehran’s WWTPs, respectively. According to these values and the population covered by Tehran’s WWTPs (3,150,000 persons/d), it is estimated that 504 m^3^/d and 346 m^3^/d of sludge will be produced and waste as excrement raw sludge, respectively, in Tehran. This high volume of sludge needs to be managed and discarded properly. The most common method of treatment for these sludge is digestion (aerobically and an aerobically). The lagooning, composting, and landfilling are the next methods of treatment. Mechanical dewatering may also be implemented as final treatment to reduce the volume of the stabilized sludge.

## Conclusions

In this study, the basic characteristic data regarding to the nine main domestic wastewater treatment plants in Tehran was assessed and analyzed. The BOD_5_ and TSS parameters of 32.96 ± 1.91, 37.31 ± 2.44 g/d.cap obtained in this study are considerably lower than the values of 40 to 50 and 50 to 60 g/d.cap, respectively, recommended by Department of Energy of Iran for the design of new treatment plants. The per capita loading of wastewater were used by designers for the design of a wastewater treatment plant in Tehran, was estimated to be 215 L/d.cap while the actual per capita collected wastewater according to the real data of treatment plants was determined to be 186.06 ± 7.85 L/d.cap. This disparity has interesting implication when these estimated values are applied to the design of new wastewater treatment plant and calculation of an equivalent population for an industrial waste. The design of new treatment plant in Tehran with a per capita of 186.06 ± 7.85 L/d.cap may lower the required capacity for the treatment of wastewater up to 40%. The results of this study reveal that the Tehran’s wastewater may be classified as highly degradable, but during recent decades the Biodegradability Index has been reduced up to 15%. This may be resulted from this fact that over the past 15-20 years, a wide range of diverse industrial synthetic detergents have been produced and extensively used for cleaning and disinfection purposes and then discharged into sewage systems. According to the PDC of BOD_5_, Tehran was found to be with lower priority of BOD_5_ discharge reduction from domestic wastewater. It is estimated that 504 m^3^/d and 346 m^3^/d of sludge will be produced and waste as excrement raw sludge, respectively, in Tehran.

In conclusion, the use of new revised per capita parameters obtained in this study could help in designing more efficient treatment systems and generating more reliable data for operational control of wastewater treatment process in Tehran. However, further research on wastewater quality and quantity assessment and adequate monitoring measures are required for existing and future treatment facilities in Tehran to ensure that they comply with safe operational and environmental standards.

### Endnote

^a^Regional Organization for the Protection of the Marine Environment.

## Nomenclature

Nomin; the nominal capacity of WWTP (m^3^/month)

Collect; the collected volume of wastewater (m^3^/month)

Treat; the treated volume of wastewater (m^3^/month)

ProSlu; the produced sludge (m^3^/month)

ExcSlu; the excess sludge (m^3^/month)

CurentPop; the current covered population

Tin; the Influent temperature (C°)

pHin; the Influent pH

BODin; the Influent BOD_5_ (mg/L)

CODin; the influent COD (mg/L)

TSin; the influent total solids (mg/L)

TSSin; the influent suspended solids (mg/L)

TDSin; the influent dissolved solids (mg/L)

Pin; the influent phosphorous (mg/L)

OrNin; the influent organic nitrogen (mg/L)

TKNin; the influent total Kjeldahl nitrogen (mg/L)

Tout; the effluent temperature (C°)

pHout; the effluent pH

BODout; the effluent BOD_5_ (mg/L)

CODout; the effluent COD (mg/L)

TSout; the effluent total solids (mg/L)

TSSout; the effluent suspended solids (mg/L)

TDSout; the effluent dissolved solids (mg/L)

Pout; the effluent phosphorous (mg/L)

OrNout; the effluent organic nitrogen (mg/L)

TKNout; the effluent total Kjeldahl nitrogen (mg/L)

GCPD_Slu_E_; the generation per capita per day of excess sludge (L sudge/d.cap)

GCPD_Slu_P_; the generation per capita per day of produced sludge (L sludge /d.cap)

GCPD_TS_; the generation per capita per day of total solids (g /d.cap)

GCPD_OrgN_; the generation per capita per day of organic nitrigen (g /d.cap)

GCPD_TDS_; the generation per capita per day of total dissolved solids (g /d.cap)

GCPD_P_; the generation per capita per day of total phosphorous (g /d.cap)

GCPD_TKN_; the generation per capita per day of total kjeldahl nitrogen (g /d.cap)

GCPD_TSS_; the generation per capita per day of total suspended solids (g /d.cap)

GCPD_COD_; the generation per capita per day of chemical oxygen demand (g /d.cap)

GCPD_BOD5_; the generation per capita per day of biological oxygen demand (g /d.cap)

(BOD_5_/COD)_out_; the biodegradability index of effluent wastewater

(BOD_5_/COD)_in_; the biodegradability index of influent wastewater

Tret_PerP_; the per capita per day of treated wastewater (L/d.cap)

Coll_PerP_; the per capita per day of collected wastewater (L/d.cap)
